# THE INTERACTION BETWEEN SHORT SLEEP AND EXERCISE FREQUENCY WITH HEART RATE VARIABILITY: CROSS-SECTIONAL STUDY

**DOI:** 10.1093/geroni/igae098.3745

**Published:** 2024-12-31

**Authors:** Taylor Fein, Muhammad Thalil, Soomi Lee

**Affiliations:** Pennsylvania State University, Rock Hill, New York, United States; Pennsylvania State University, State College, Pennsylvania, United States; The Pennsylvania State University, University Park, Pennsylvania, United States

## Abstract

Exercise has been shown to be a powerful tool that can improve cardiovascular health. Both exercise and adequate sleep have been associated with increased heart rate variability (HRV), while insufficient amounts of both are associated with decreased HRV. Yet, the combined influence of exercise and sleep on HRV is less clear. This study explores if exercise levels and sleep duration interact to be associated with HRV. 391 adults (Mage=57yrs) from the Midlife in the United States Biomarker study 2004-2009 provided sleep actigraphy, electrocardiogram (ECG) HRV measurements and answered questions on exercise habits. Participants were grouped as short (< 6 hours) or adequate sleepers (≥ 6 hours) and inadequate (< 150 minutes per week) or adequate exercise frequency (≥150 minutes per week) based on CDC guidelines. ANCOVA analysis adjusted for demographic and health-related covariates was used to examine the interaction between sleep duration and exercise frequency on HRV. There were no significant individual associations of sleep duration or exercise frequency with HRV, however combined associations were found. For individuals with less than 6 hours of sleep, those with inadequate exercise frequency had significantly lower HF-HRV (B=-0.59, SE = 0.21, p=0.005) and RMSSD (B=0.29, SE = 0.10, p=0.003) compared to those with adequate exercise. Among adequate sleeper’s, no significant differences in HRV were observed between each exercise group. Findings suggest that for those with short sleep, lack of exercise may exacerbate the negative consequences of short sleep on HRV. Future studies should explore whether and how exercise intensity may change this relationship.

